# Prospective Evaluation of Adverse Drug Reactions in Hospitalized Older Adults in Ethiopia

**DOI:** 10.3390/jpm15060227

**Published:** 2025-06-01

**Authors:** Mengist Awoke Yizengaw, Behailu Terefe Tesfaye, Dula Dessalegn Bosho, Gebremichael Tesfay Desta, Mohammed S. Salahudeen

**Affiliations:** 1 School of Pharmacy, Faculty of Health Science, Jimma University, Jimma P.O. Box 378, Ethiopia; mengist93@gmail.com (M.A.Y.); behailu.terefe@ju.edu.et (B.T.T.); dula.dessalegn@ju.edu.et (D.D.B.); gebremichael.tesfay@ju.edu.et (G.T.D.); 2 School of Pharmacy and Pharmacology, College of Health and Medicine, University of Tasmania, Hobart, TAS 7001, Australia

**Keywords:** adverse drug reaction (ADR), severity, preventability, older adults

## Abstract

**Background:** Older adults are vulnerable to adverse drug reactions (ADRs), particularly in low-income settings, yet data on ADR prevalence in Africa, including Ethiopia, remain limited. **Objective:** This study aimed to evaluate the incidence, severity, and preventability of ADRs among hospitalized older adults, as well as all-cause inpatient mortality. **Methods:** A cross-sectional observational study was conducted at Jimma Medical Center, located in Jimma town, Ethiopia, from 6 September 2021 to 26 December 2022. The study participants were older adults (n = 162) admitted to the medical wards. ADRs were assessed using the Naranjo ADR probability scale, severity was classified according to the modified Hartwig and Siegel criteria, and preventability was determined using the Schumock and Thornton criteria. **Results:** The median age of participants was 65 years (interquartile range: 60–70). During their hospital stay, 84 patients (51.9%) experienced at least one ADR. A total of 123 ADRs (76 ADRs per 100 admissions) were captured. Most ADRs (93.5%) were classified as mild to moderate in severity, and 84.5% (n = 105) were considered preventable. Endocrine and metabolic systems (48.8%) and diuretics (43.9%) were the most frequently affected organ systems and drug class linked to ADRs, respectively. Furosemide (41.5%) and aspirin (10.6%) were the most frequently implicated medications, commonly causing hypokalemia (35.3%) and dyspepsia (53.8%), respectively. The observed all-cause in-patient mortality rate was 6.8% (5 deaths per 1000 patient-days). The use of potentially inappropriate medications (PIMs) (aOR: 4.747, *p* = 0.003) and presence of digestive system disorders (aOR: 8.784, *p* = 0.038) were associated with an increased risk of ADRs, while a history of recent traditional medicine use (aOR: 0.285, *p* = 0.042) was linked to a lower risk. **Conclusions:** More than half of the hospitalized older adults experienced ADRs, most of which were mild to moderate in severity and considered preventable. Regular medication review for screening and minimizing PIM use in older adults may play a crucial role in lowering ADR occurrence. The borderline but statistically significant association between a history of traditional medicine use and lower occurrence of ADRs requires cautious interpretation and further investigation to explore possible explanations. Nearly seven deaths per hundred hospitalized patients were recorded.

## 1. Introduction

An adverse drug reaction (ADR) is defined as an undesirable or harmful reaction that occurs after the administration of a drug or combination of drugs under normal conditions of use [1]. An ADR causes physical harm, prolonged hospital stays, increased healthcare expenditure, reduced quality of life, and mortality [2,3,4,5,6,7]. This burden disproportionately affects vulnerable populations, such as older adults [8]. For instance, ADRs account for about 6.0% of all 30-day hospital readmissions in older adults [9].

The propensity to ADRs in older adults (aged 65 years and above) is associated with their vulnerability to multimorbidity, polypharmacy, and age-related decline in organ functions, leading to adverse clinical, economic, and humanistic impacts [10]. Various studies across countries recorded ADR prevalence ranging from 9.8% in Australia [3] to as high as 69% in Uganda [11]. Additionally, ADR incidence in older adults during hospitalization is profoundly high, and reaching up to 76.1% [12], with nearly one-third of ADRs classified as severe [5]. Interestingly, about 55–95% of the ADRs in older adults are considered preventable through regular medication reviews and adherence to prescribing guidelines tailored to older adults [5,13,14].

Potentially modifiable risk factors, including extended hospital stays, emergency hospital admissions, smoking status, polypharmacy, use of potentially inappropriate medications (PIMs), and concurrent use of complementary or alternative medicines were among the specific targets to lower the ADR occurrence rate [5,15].

In low- and middle-income countries (LMICs), including Ethiopia, pharmacovigilance systems often face structural and operational challenges that limit ADR detection and reporting, including lack of awareness, limited training, and weak regulatory systems [16,17]. This is supported by a systematic review showing significantly higher ADR rates in LMICs (29%) compared to high-income countries (19%) [5].

In Ethiopia, despite a growing proportion of older adults, only a single study assessed ADR prevalence among hospitalized older adults (31.1%) [18]. Studies determining the extent and assessing the characteristics of ADRs, including their potential preventability, will have significant benefits, such as alerting clinicians and policy makers in designing context-tailored intervention modalities that will reduce the incidence of ADRs. This research aimed to determine the incidence, severity, and preventability of ADRs in hospitalized older adults and describe the all-cause inpatient mortality rate in southwest Ethiopia.

## 2. Methods

### 2.1. Study Design, Setting, and Period

This cross-sectional observational study was conducted at Jimma Medical Center (JMC) from 6 September 2021 to 26 December 2022, a major teaching and referral hospital located in Jimma, Southwest Ethiopia, approximately 352 km from Addis Ababa. JMC has 16 departments with over 970 beds, including around 100 beds in the medical wards, and employs over 1600 staff members. The hospital serves around 16,000 inpatients and 220,000 outpatients annually, covering a catchment population of about 20 million people [19]. In the study setting, although ward pharmacists are already assigned, pharmacist-led medication review is not well integrated in its current state.

### 2.2. Eligibility Criteria

This study included all hospitalized patients aged 60 years and over admitted to the medical wards of JMC during the study period. All eligible patients were enrolled upon admission to the ward and followed until discharge. In contrast, older adults discharged within 24 hr of hospital admission, readmitted patients, and patients who were unable to provide consent (e.g., patients with aphasia) to participate were excluded from the study.

### 2.3. Sample Size

The sample size was calculated using a single population proportion formula with a 5% margin of error, assuming a 50% prevalence of ADRs among hospitalized older adults. Based on the previous year’s admissions of older adults at medical wards of JMC (2019–2020), which was 497, a corrected final sample size of 162 participants was determined. Consecutive sampling was used to recruit eligible participants until the required sample size was achieved.

### 2.4. Data Collection

The data collection tool was designed after reviewing the relevant literature. The tool comprised of sociodemographic, clinical, laboratory, and medication-related variables, and outcomes. Data were collected from patient medical charts, laboratory results, patient/caregiver interviews, and clinicians responsible for patient care.

Sociodemographic characteristics such as gender, age, residence, educational level, occupation, cigarette smoking, alcohol consumption, khat chewing, living arrangement, and activities of daily living (ADLs) were recorded at admission. Khat (*Catha edulis*) is a plant whose leaves and shoots are consumed by people for their stimulant effects [20]. ADLs were assessed using the Katz Index of Independence in ADL, a tool for assessing the functional health status (disability) of older individuals [21].

Clinical assessments included cognitive status at admission, the Charlson Comorbidity Index (CCI), laboratory tests (serum creatinine, sodium level, potassium level, liver and renal function tests, and coagulation profiles), medical history, and length of stay (LOS). Vital signs and anthropometric measures were also recorded. The psychological condition of each patient on admission was objectively assessed using the shortened form of the Geriatric Depression Scale (GDS), which comprised 15 items [22]. The tool was previously used to measure the cognitive functioning of older adults in Ethiopia [23].

Medication-related information was gathered, including a history of traditional medicine use, ADRs at admission, and the number and type of medications at admission, during hospitalization, and discharge. The presence or absence of PIM use was assessed using the 2019 American Geriatrics Associations Beers Criteria [24,25]. For in-patient mortality, the time when the event occurred was recorded.

### 2.5. ADR Assessment

To identify specific ADRs, a range of definitions were used. Drug-induced hepatotoxicity was defined as an increase in AST or ALT levels of at least twice the upper limit of normal. A decrease in the estimated glomerular filtration rate (eGFR) to less than 60 mL/min/1.73 m^2^ or a rise in serum creatinine of 0.3 mg/dL from baseline or reaching 1.5 mg/dL was used to define renal failure. Hypotension was defined as systolic blood pressure under 90 mmHg or diastolic blood pressure below 60 mmHg [26]. A random plasma glucose level of less than 55 mg/dL or 3 mmol/L, with or without clinical symptoms, was recorded as hypoglycemia [27]. Drug-induced constipation was noted, when no bowel movement for at least 72 h was reported [28,29]. Hyperchloremia was recorded when the plasma concentration of chloride exceeded 105 to 115 mmol/L. Serum potassium values of less than 3.6 mmol/L and more than 5.5 mmol/L were classified as hypokalemia and hyperkalemia, respectively. Hypocalcemia was recorded when the total calcium level was less than 8.8 mg/dL, and hypercalcemia with levels greater than 10.7 mg/dL. When the serum sodium level was less than 135 mEq/L, hyponatremia was recorded, whereas serum sodium levels greater than 145 mEq/L were characterized as hypernatremia [30]. Thrombocytopenia was documented as a platelet count less than 150000/microliter [31]. When the breathing rate per minute was less than 12 and greater than 20, bradypnea and tachypnea were recorded, respectively. Bradycardia and tachypnea were documented with a resting heart rate of less than 60 or more than 100 beats per minute, accordingly [32].

The Adverse Drug Event (ADE) Trigger Tool and the medication module of the Institute for Healthcare Improvement Global Trigger Tool for measuring ADEs were used to facilitate the manual chart reviews and to enhance the detection of ADRs [33,34]. For example, an older adult taking vitamin K would be assessed on if they are taking vitamin K in response to a prolonged prothrombin time (PTT) or international normalized ratio (INR). If either laboratory value is high, the specific patient’s chart was thoroughly reviewed for evidence of bleeding. Laboratory reports were checked for a fall in hematocrit or for guaiac-positive stools and the progress notes for evidence of bruising or gastrointestinal bleeding. The patient was also checked for diagnosis of hemorrhagic stroke or other internal bleeding that might have occurred. Stools have shown validity and reliability in assessing ADR in adults [35] and were used in a previous study in Ethiopia [36]. ADR causality was evaluated using the Naranjo ADR probability scale (definite, probable, possible, doubtful) [37], which has good sensitivity, reliability, content, and concurrent validity when used in post-marketing drug surveillance [37]. Two clinical pharmacists and a nurse were trained on the data collection tool and procedure. Each ADR was assessed by two clinical pharmacists independently daily, and the final judgment was made after verification with in-charge clinicians.

The 7-item modified Hartwig and Siegel severity assessment scale was used to assess the severity of ADRs, with mild, moderate, and severe categories [38]. The preventability of ADRs was determined using the 7-item Schumock and Thornton preventability assessment scale, which are divided into definitely preventable, probably preventable, and non-preventable [39]. The medications used during hospital stay were classified using the 2024 World Health Organization (WHO) Anatomical Therapeutic Chemical (ATC) system [40]. The International Statistical Classification of Diseases for Mortality and Morbidity Statistics (ICD-11 MMS) was used to categorize patient diagnoses [41].

Data completeness and accuracy were verified daily. Data collection tools were pre-tested on 17 participants to check their validity and were adjusted accordingly. Data entry involved double-entry and cross-verification to minimize errors, with secure data storage ensuring confidentiality.

### 2.6. Data Analysis

Data were entered into Epi data version 4.2.0 and analyzed using STATA version 17.0. Prior to data analysis, the dataset was cleaned in STATA by computing descriptive statistics that summarize the number of observations, unique values, and missing values, ensuring the data were accurate, consistent, and complete. Categorical variables were appropriately coded and recorded. Missing data, specifically BMI (n = 8), were handled by replacing them with mean values. Descriptive statistics (frequency, percentage, mean, SD, median, IQR) summarized categorical and continuous variables. The incidence of ADRs per 100 admissions was computed, while the all-cause inpatient mortality rate per 1000 patient-days was calculated. Bivariate logistic regression was employed to identify the relationship between ADR occurrence and predictors. Variables with a *p* < 0.25 were then included in a stepwise multivariate logistic regression model to adjust the odds ratios (ORs) for potential confounders. The effect size was presented using an OR with a 95% confidence interval (CI). Multicollinearity was checked using a variance inflation factor (VIF), and none of the variables achieved a VIF > 5 (the maximum was 2.05). The Hosmer–Lemeshow test was computed to check the goodness-of-fit of the final model, in addition to sensitivity and specificity. Subgroup analysis was performed on commonly reported variables from the GerontoNet ADR risk score [42] and related literature in relation to ADR occurrence. A *p* value < 0.05 was used to declare statistical significance.

## 3. Results

### 3.1. Study Participant’s Enrollment Information

A total of 176 older adults were assessed for eligibility. In total, 14 participants were excluded: 12 due to readmission during the study period, and 2 due to aphasia. Consequently, 162 older adults were included in the final analysis.

### 3.2. Characteristics of the Study Participants

The median age of the participants was 65 years (IQR: 60–70), and the majority were males (n = 134, 82.7%). More than half of the participants (n = 85, 52.5%) lived with their spouse and children (Table 1).

### 3.3. Clinical Characteristics and Patient Discharge Outcome

Overall, 105 participants (64.5%) had at least one chronic medical condition, and about two-thirds had no hospitalization history in the past year. The median CCI score was 4 (IQR: 3–5), and the median number of medications prescribed per patient was 6 (IQR: 4–7). Eleven patients (6.8%) died during their hospital stay. The total analysis time at risk was 2177 days, accordingly, the incidence rate of inpatient mortality was 0.005 (5 per 1000 patient-days) (Table 2).

### 3.4. Incidence and Characteristics of ADR

During the hospital stay, more than half (n = 84, 51.9%) of the participants experienced at least one ADR. A total of 123 ADRs were detected, implying nearly 1.5 ADRs per patient and an overall incidence of 76 ADRs per 100 hospital admissions. According to the Naranjo algorithm, about half (48.8%, n = 60) of the ADRs were categorized as possible, and only 4 (3.2%) were classified as definite. Most ADRs (93.5%, n = 115) were mild to moderate in severity, and a substantial proportion (84.6%, n = 104) were deemed preventable (Table 3).

**Figure 1:**
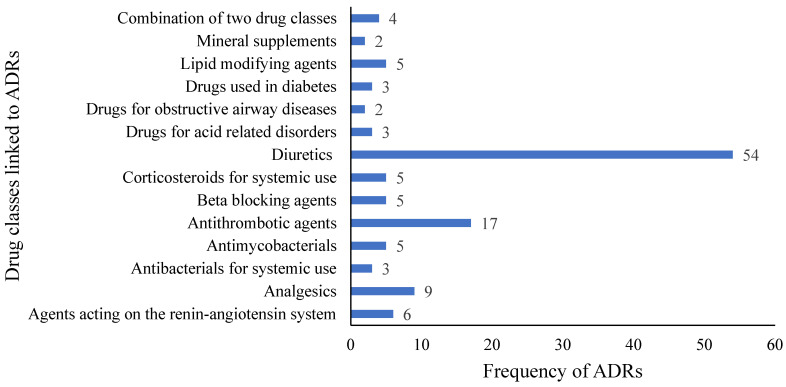
Frequency of ADRs in terms of drug classes.

The most frequently implicated drug class with ADRs was diuretics (43.9%, n = 54) followed by antithrombotics/antiplatelets (17, 13.8%) (Figure 1).

Furosemide accounted for about 94.4% of the ADRs ascribed to diuretics, while aspirin is responsible for 76.4% of the ADRs from antithrombotics/antiplatelets. Furosemide was most frequently associated with hypokalemia (13.8%, n = 17), and aspirin was primarily linked to dyspepsia (53.8%, n = 7). The three most common ADRs were hypokalemia (17.9%, n = 22), hyponatremia (17.9%, n = 22), and hypotension (10.6%, n = 13). The endocrine and metabolic system (48.8%) was the most frequently affected organ system followed by gastrointestinal (22.8%) and cardiovascular (13.8%) systems (Table 4).

In bivariate logistic regression analysis, the number of inpatient medications and PIM use were strongly associated with ADR occurrence (*p* < 0.001)**.** Seventeen variables met the threshold for inclusion in the multivariate analysis. In the final multivariate model, PIM use remained significantly associated with an increased likelihood of ADR occurrence, with users having nearly five times higher odds compared to non-users (aOR: 4.75, 95% CI: 1.68–13.39, *p* = 0.003). Patients diagnosed with digestive system disorder also showed an increased ADR risk (aOR: 8.78, 95% CI: 1.13–68.13, *p* = 0.038). History of traditional medicine use was statistically associated with a lower occurrence of ADRs (aOR: 0.29, 95% CI: 0.09–0.96, *p* = 0.042); however, this finding requires cautious interpretation and further investigation to explore possible explanations. The Hosmer–Lemeshow test indicated good model fit (*p* = 0.367), with sensitivity and specificity values of 75.0% and 73.1%, respectively (Table 5).

In the regression model, the contribution of potential interaction terms, polypharmacy and PIM use, was found statistically non-significant (*p* = 0.390). Furthermore, the reduced model was better than the full model for the study’s data.

According to the GerontoNet ADR risk score [42], in addition to the number of drugs, patients with heart failure, liver disease, history of ADR, and renal failure are placed at high risk for ADRs. In our study, there were 51 (35.2%), 5 (3.1%), 16 (9.9%), and 5 (3.1%) patients with diagnosis of heart failure, chronic liver disease, history of ADRs, and chronic kidney disease, respectively. In total, 104 (64.2%) were aged 60–69, while those aged 70 and above were 58 (35.8%). In patients with heart failure, the data indicate a significantly lower rate of ADRs compared to those without heart failure (48.8% versus 51.9%, *p* < 0.0001). However, no statistically significant association was observed in patients with or without CLD (4.8% versus 95.2%, *p* = 0.369), history of ADR (10.7% versus 89.3%, *p* = 0.711), and CKD (3.6% versus 96.4%, *p* = 1.000). The median length of hospital stays in patients with (10 days) and without (9 days) ADRs was not significantly different (*p*= 0.2243). Similarly, the incidence of ADR in patients aged 60–69 (61.9%) versus greater than or equal to 70 years (38.1%) was not statistically significant (*p* = 0.528).

## 4. Discussion

This observational study, the first of its kind in southwest Ethiopia, provides valuable insights into the incidence, severity, and preventability of ADRs among hospitalized older adults.

Over half (51.9%, 76 per 100 admissions) of the study participants experienced at least one ADR during their hospital stay, which is nearly similar with a report from Uganda (48.9%) [11], potentially indicating similarities in prescribing patterns, polypharmacy use, and healthcare practices in the care of older adults in sub-Saharan Africa. Nevertheless, the magnitude of ADRs observed in the current study exceeds those reported in previous prospective studies involving hospitalized older adults, including those conducted in high-income countries such as Chile (24.5%) [43], Europe (21.6%) [44], Japan (15.4%) [45], and Australia (9.8%) [3]. This disparity may reflect differences in healthcare delivery, prescribing patterns, and resource availability across settings. In high-income countries, geriatric care is more structured and multidisciplinary, with greater adherence to clinical guidelines, utilization of electronic decision support systems, and routine medication reviews by clinical pharmacists or geriatricians. This may have contributed to the lower ADRs in older adults in these regions. Likewise, studies from low-middle income countries, such as India (25–32.2%) [46,47,48], Pakistan (10.5%) [49], and Northern Ethiopia (31.1%) [18] reported a lower incidence rate. This variation may stem from the methodological differences in ADR causality assessment tools (WHO Uppsala Monitoring Center vs. Naranjo) and classification. The present study included all ADR categories as per Naranjo’s algorithm. The high incidence of ADRs suggests a need to evaluate prescribing practices, monitoring protocols, and patient safety systems in the local healthcare context.

Several studies across countries consistently reported that a significant proportion of ADRs are preventable. For instance, findings from Ireland (71.1%) [2], India (48.4%) [48], Uganda (45.2%) [50], Northen Ethiopia (61.9%) [21], and LMICs (60%) [5] demonstrated that a substantial share of ADRs are avoidable. In this study, a notably higher proportion of ADRs (84.6%) were classified as definitely or probably preventable. This highlights a potential opportunity to reduce medication-related harm in hospitalized older adults through targeted interventions like regular medication reviews, deprescribing, and vigilant medication reconciliation [51], which should be further evaluated in the current study setting.

Our findings indicate that PIM users had a nearly fivefold increased risk of ADRs compared to non-users, which is consistent with previous studies from Uganda [11] and Australia [52]. These collectively imply the need for regular, structured interdisciplinary medication reviews, engaging both physicians and clinical pharmacists, to identify PIM, thereby reducing preventable ADRs. Although we used the Beers Criteria for identifying PIMs, alternative tools such as the STOPP/START criteria offer complementary insights, especially when considering drug–disease interactions and underprescribing risks [53]. Inclusion of these tools in future studies may enrich medication appropriateness assessments. Older patients with digestive system disorders exhibited approximately nine times the likelihood of experiencing ADRs, reflecting pathophysiological changes that alter drug absorption, metabolism, and excretion [54]. Clinically, this supports the need for careful medication management in older adults with gastrointestinal comorbidities, especially when prescribing drugs with known gastrointestinal toxicity, to mitigate ADR risks and ensure safer pharmacological treatment.

Borderline statistically significant association was found between recent history of traditional medicine use and a lower ADR risk among older adults; however, this finding requires cautious interpretation and further ethnopharmacological studies for explanation. The available evidence shows inconsistent findings; reports from Uganda [55] and Brazil [56] indicate increased ADR risks associated with traditional medicine use, while research from China [57] aligns with our observation of potentially reduced risks. While the exact mechanism remains unclear, some herbal products may indeed interact pharmacodynamically or pharmacokinetically with conventional medicines, potentially influencing ADR profiles [58]. This intriguing finding warrants further investigation to determine whether certain traditional medicines confer protective effects or simply reflect cautious prescribing behaviors by providers aware of concurrent herbal medication use.

The proportion of severe ADRs in our study (6.5%) is comparable with findings from Uganda (2.8%) [50] and Ireland (7.2%) [2], while studies from India have documented higher rates, ranging from 11% [59] to 19.3% [60]. Differences in patient recruitment settings, underlying frailty, and the assessment criteria are likely to contribute to these discrepancies. Furthermore, the high prevalence of electrolyte imbalances observed, specifically hypokalemia (17.1%) and hyponatremia (17.9%), aligns with previous studies from Italy [61] and India [59], although these rates were notably higher than in some other studies [18,62]. This variation may be related to the types of medical conditions and medications used, particularly those that affect electrolyte balance, such as diuretics and ACE inhibitors taken by the current study participants.

Our observed inpatient mortality rate (6.8%) aligns closely with previous reports from China [63], Spain [64], and Turkey [65], but is significantly lower than in studies from Pakistan [66], Cameroon [67], and Italy [68], which included more severely ill subpopulations. The discrepancies may be attributed to the differences in sociodemographic factors, clinical characteristics, underlying comorbidities, and healthcare system. This notable in-patient mortality rate emphasizes the importance of reinforcing comprehensive inpatient geriatric care to enhance patient outcomes and reduce preventable adverse outcomes.

Older patients taking furosemide experienced the highest number of ADRs, such as electrolyte disturbances and dehydration, which are especially concerning in this age group due to age-related changes in renal function and fluid regulation [69] as well as its potential for exacerbating or causing the syndrome of inappropriate antidiuretic hormone secretion [24]. A 2020 meta-analysis demonstrated that diuretics (19.8%) was the most frequently identified medication class linked to ADRs [13], which is consistent with the findings of the present study, where diuretics was the most commonly implicated medication class in ADRs (43.9%), with furosemide accounting for the majority (41.5%) of diuretic-related cases. The Beers Criteria recommends cautious use of furosemide by initiating a low dose (e.g., 20 mg instead of 40 mg), titrating slowly while monitoring renal function and electrolytes, and avoiding concurrent nephrotoxic drugs to minimize their harm [24].

In our study, antithrombotic/antiplatelets. (13.8%) was the second most common drug class implicated in ADRs, with aspirin alone accounting for 10.6%, the second most frequently involved agent, which closely aligns with findings from a prospective study in Northern Ethiopia (12.4%) [18] and a systematic review and meta-analysis report (12.2%) [13]. This burden may be attributed to age-related change in drug pharmacokinetics, decline in renal function, polypharmacy, and drug–drug and drug–disease interactions, which significantly increased the risk of ADRs [70]. Enhancing clinical pharmacist involvement in ward rounds and utilizing risk assessment tools may play a critical role in minimizing antithrombotic-associated ADRs in hospital settings.

In the current study, the endocrine and metabolic system (48.8%) and gastrointestinal system (22.8%) were the most frequent organ systems affected by ADRs among hospitalized older adults. This finding is slightly different compared with a report from Northern Ethiopia, where gastrointestinal tract (28.92%) followed by the cardiovascular system (19.01%) were the frequently affected organs [18]. In addition, a systematic review and meta-analysis revealed fluid and electrolyte disturbances (17.3%) and gastrointestinal disorders (13.3%) among the most organs affected [13]. This discrepancy may be explained by variations in patient demographics, comorbid conditions, definition criteria, and associated prescribing patterns across the study population. Close monitoring of blood glucose, electrolytes, and renal function, coupled with careful clinical assessment for metabolic imbalances and gastrointestinal disturbances, alongside context-specific pharmacovigilance, may be an effective way for preventing and managing ADRs in our study settings.

The lower ADR rate observed in heart failure patients (48.8% vs. 51.9%) may be partly due to under-recognition of ADRs in this population, as symptoms like fatigue, dizziness, and hypotension overlap with the clinical features of heart failure, leading to under-rereporting. Additionally, these patients often undergo close monitoring of renal function, electrolytes, and drug levels, which may help prevent ADRs. Confounding factors such as differences in comorbidity profiles and patterns of medication exposure may also have contributed to the observed difference.

### Study Limitations

While this study provides important prospective data on ADR incidence, potential preventability, and risk factors among hospitalized older adults in Ethiopia, several key aspects commonly reported in the ADR literature were not captured.

First, the time to the onset of the ADRs was not assessed, which limits our understanding of temporal patterns. Additionally, the seriousness of ADRs based on the WHO’s definition has not been determined; future studies should address this gap.

This study also lacked a cost analysis component, preventing insights into the economic burden of ADRs, which is increasingly relevant in low-resource settings. Preventable ADRs contribute significantly to healthcare costs, as they often result in prolonged hospitalizations, emergency department visits, additional treatments, and increased use of healthcare resources [71,72,73]. A systematic review published in 2018 indicated that ADRs are responsible for direct healthcare costs ranging from EUR 63.8 to EUR 9015 per hospitalization [6]. Another study revealed that the total direct costs for older adults with ADRs (EUR 3501) are four times higher than those for individuals without ADRs (EUR 929) [72]. Additionally, hospital admissions due to ADRs incur higher average costs (EUR 9538) than non-ADR admissions (EUR 9828), with preventable ADRs accounting for 69% (EUR 3907) of the total incremental costs associated with ADRs [71]. These preventable ADRs draw particular attention as their financial burden and adverse clinical outcomes are potentially avoidable through appropriate interventions. Therefore, economic impact focused studies are critical to address this gap in the current study setting.

The Naranjo algorithm has demonstrated good sensitivity, reliability, and validity for use in post-marketing drug surveillance and clinical settings. Similarly, the ADR trigger tool employed in this study has shown validity and reliability in adult populations. However, our study did not include dechallenge/rechallenge data, and neither the Naranjo algorithm nor the ADR trigger tool was locally validated against alternative ADR detection methods. This limitation highlights the need for future local research to evaluate the psychometric properties of these pharmacovigilance tools in comparable healthcare settings.

Furthermore, this study did not assess ADR-related post-discharge outcomes (e.g., mortality and rehospitalization), implying longitudinal follow-up future studies. The role of healthcare professionals in identifying and mitigating ADRs is also unaddressed in this study. Hence, integrating a qualitative component or survey in future studies to evaluate clinical practice factors is necessary.

Even though the ADR assessors and data collectors were not part of the investigation team, a measure that could help reduce observer bias, this benefit may be diminished by the lack of blinding for both groups regarding the outcomes. Additionally, we were unable to assess inter-rater reliability since the ADR assessments for individual patients were conducted by one of the two data collectors.

The marginal statistically significant association between recent traditional medicine use history and lower ADR occurrence precludes a stronger conclusion; as a result, cautious interpretation of this finding is recommended, and mechanistic or ethnopharmacological studies are required to better explain the relationship.

Lastly, this is a single-center study, which may limit the generalizability of the findings to other healthcare settings or regions. Additionally, it did not assess the long-term impact of ADRs on patient outcomes. We recommend that future multicenter studies be conducted to address these limitations, thereby strengthening the evidence base for prevention strategies and enhancing patient safety outcomes.

## 5. Conclusions

More than half of the hospitalized older adults experienced ADRs, most of which were mild to moderate in severity and considered preventable. Regular medication review for screening and minimizing PIM use in older adults may play a crucial role in lowering ADR occurrence. The borderline but statistically significant association between a history of traditional medicine use and lower occurrence of ADRs requires cautious interpretation and further investigation to explore possible explanations. Nearly seven deaths per hundred hospitalized patients were recorded.

## Figures and Tables

**Table 1:** The study participants’ sociodemographic and behavioral characteristics (N = 162).

Sociodemographic and Behavioral Information		Frequency (n, %)
Age (in years)	Median (IQR)	65 (60,70)
Sex	Male	134 (82.7)
	Female	28 (17.3)
Residence	Rural	33 (20.4)
	Urban	129 (79.6)
Marital status	Never married	1 (0.6)
	Married	134 (82.7)
	Divorced	8 (4.9)
	Widowed	19 (11.8)
Level of education	Cannot read and write	120 (74.1)
	Informal education	33 (20.4)
	Primary education (1–8 grade)	6 (3.7)
	College and above	3 (1.9%)
Currently working	Yes	53 (32.7)
	No	109 (67.3)
Employment status	Retired	20 (12.4)
	Employed	1 (0.6)
	Private work	52 (32.1)
	Non-employed	89 (54.9)
Financial dependence	Dependent	34 (21)
	Independent	128 (79)
Alcohol consumption	Never	116 (71.6)
	Previously	44 (27.2)
	Current	2 (1.2)
Smoking	Never	121 (74.7)
	Ex-smoker	39 (24.1)
	Current smoker	2 (1.2)
Khat chewing	Never	45 (27.8)
	Previously	105 (64.8)
	Current	12 (7.4)
Traditional medicine use history	Yes	21 (13)
	No	141 (87)
Mode of living	Live with spouse and children	85 (52.5)
	Live with spouse	41 (25.3)
	Live with children	29 (17.9)
	Live alone	7 (4.3)
Activities of daily living (ADL)	Median (IQR) Katz Score	3.5 (0,6)
	Dependent	65 (40.1)
	Partially dependent	51 (31.5)
	Fully independent	46 (28.4)
BMI, kg/m^2^	Median (IQR)	19.5 (17.8, 20.7)
	Underweight (less than 18.5)	46 (28.4)
	Normal (18.5 to <25)	107 (66.1)
	Overweight (25.0 to <30)	9 (5.6)

BMI—Body Mass Index; IQR—interquartile range; kg/m^2^—kilogram per meter square.

**Table 2:** Clinical and medication characteristics and in-patient discharge outcomes of the study participants (N = 162).

Clinical, Medication, and Discharge Status Information	Frequency (n, %)
Patients with chronic medical condition	105 (64.8)
Hospitalization in the last 1 year before the study period	
None	109 (67.3)
One	49 (30.3)
Two and above	4 (2.5)
Psychological condition on admission (GDS score)	
No psychological problems (0 to 4)	34 (21)
Mild dementia/depression (5 to 9)	96 (59.3)
Severe dementia/depression (10 to 15)	32 (19.8)
Diagnoses according to ICD-11 classification	
Certain infectious or parasitic diseases	20 (12.4)
Neoplasms	3 (1.9)
Diseases of the immune system	5 (3.1)
Endocrine, nutritional or metabolic diseases	35 (21.6)
Mental, behavioral or neurodevelopmental disorders	2 (1.23)
Diseases of the nervous system	21 (13)
Diseases of the circulatory system	112 (69.1)
Diseases of the respiratory system	70 (43.2)
Diseases of the digestive system	12 (7.4)
Diseases of the skin	1 (0.6)
Diseases of the blood or blood-forming organs	33 (20.4)
Diseases of the genitourinary system	37 (22.8)
Symptoms, signs, or clinical findings, not elsewhere classified	13 (8)
Diseases diagnosed per patient, median (IQR)	3 (3, 4)
Charlson Comorbidity Index score, median (IQR)	4 (3, 5)
Hospital stays in days, median (IQR)	10 (6, 14)
Previous ADR history	16 (9.9)
Inpatient medications class according to ATC	
A: Alimentary tract and metabolism	90 (55.6)
B: Blood and blood-forming organs	98 (60.5)
C: Cardiovascular system	120 (74.1)
H: Systemic hormonal preparations	32 (19.8)
J: Anti-infective for systemic use	110 (67.9)
M: Musculoskeletal system	2 (1.2)
N: Nervous system	40 (24.7)
P: Antiparasitic products	1 (0.6)
R: Respiratory system	29 (17.9)
V: Various agents	4 (2.5)
Medications per patient, median (IQR)	6 (4, 7)
Patient discharge outcome	
Alive	151 (93.2%)
Death	11 (6.8%)

ATC—Anatomical Therapeutic Chemical; GDS—Geriatric Depression Scale; ICD—International Classification of Diseases 11th Revision; IQR—interquartile range.

**Table 3:** Incidence, severity, and preventability of ADRs (N = 162).

Incidence and Category of ADR	Frequency (n, %)
Participants who experienced at least one ADR	84 (51.9)
ADRs per patient	
One	55 (65.5)
Two	20 (23.8)
Three and above	9 (10.7)
Total number of ADRs captured	123
Minimum, Maximum	1, 4
Median (IQR)	1 (1–2)
Causality per Naranjo ADR Probability Scale	
Definite	4 (3.2)
Probable	46 (37.4)
Possible	60 (48.8)
Doubtful	13 (10.6)
ADR Severity per Hartwig and Siegel Assessment Scale	
Mild	66 (53.7)
Moderate	49 (39.8)
Severe	8 (6.5)
Preventability per Schumock and Thornton Assessment Scale	
Definitely preventable	61 (49.6)
Probably preventable	43 (35)
Not preventable	19 (15.4)

ADRs—adverse drug reactions; IQR—interquartile range.

**Table 4:** Profile of the incident ADRs classified by the organ systems affected (N = 123).

Organ System Affected	ADR, n (%)	Specific ADR (n)	Specific Drug Causing ADR	Number of ADRs	Naranjo Probability of Causality	Preventability of the ADR	Severity of the ADR
Cardiovascular system	17 (13.8)	Hypotension (13)	Atenolol	1	Possible	Definite	Moderate
			Furosemide	8	Probable (4), possible (4)	Definite (5), probable (3)	Moderate (7), severe (1)
			Metoprolol tartarate	3	Probable (2), possible (1)	Definite (1), probable (2)	Moderate
			Tramadol	1	Possible	Definite	Moderate
		Tachycardia (4)	Furosemide	3	Probable (2), possible (1)	Definite (2), probable (1)	Mild (1), moderate (2)
			Salbutamol/Albuterol	1	Possible	Not preventable	Mild
Endocrine and metabolic system	60 (48.8)	Hyperchloremia (4)	Aspirin	3	Probable (3)	Not preventable (3)	Mild
			Paracetamol/Acetaminophen	1	Possible	Not preventable	Mild
		Hyperglycemia (4)	Dexamethasone	3	Probable (3)	Definite (1) Probable (1) Not preventable (1)	Mild
			Hydrocortisone	1	Probable	Not preventable	Mild
		Hyperkalemia (6)	Potassium chloride	1	Definite	Definite	Moderate
			Enalapril	3	Definite (1), probable (2)	Definite (1) probable (2)	Mild
			Enalapril + Spironolactone	2	Probable (2)	Definite (2)	Mild
		Hypocalcemia (1)	Furosemide	1	Probable	Definite	Severe
		Hypoglycemia (2)	Doxycycline	1	Possible	Definite	Severe
			Metoprolol tartarate	1	Possible	Definite	Mild
		Hypokalemia (21)	Furosemide	17	Definite (1), probable (9), possible (7)	Definite (11), probable (5), not preventable (1)	Mild (11), moderate (6)
			Insulin	3	Probable	Definite (1), probable (2)	Mild (2), moderate (1)
			Salbutamol/Albuterol	1	Possible	Definite	Moderate
		Hyponatremia (22)	Atorvastatin	1	Possible	Definite	Mild
			Captopril	1	Possible	Probable	Mild
			Enalapril	1	Possible	Probable	Mild
			Furosemide	8	Probable (2), possible (6)	Definite (2), probable (6)	Mild (7), severe (1)
			Morphine	2	Possible	Definite (1), probable (1)	Mild
			Omeprazole	2	Possible (1), doubtful (1)	Definite (1), not preventable (1)	Mild
			Spironolactone	3	Definite (1), possible (2)	Probable (2), not preventable (1)	Moderate
			Tramadol	1	Doubtful	Not preventable	Mild
			Unfractionated heparin	1	Possible	Probable	Mild
			Insulin + Ciprofloxacin	1	Doubtful	Definite	Mild
			Omeprazole + Enalapril	1	Possible	Probable	Mild
Gastrointestinal system	28 (22.8)	Constipation (3)	Morphine	2	Probable (1), possible (1)	Definite (1), probable (1)	Moderate (1), severe (1)
			Tramadol	1	Probable	Definite	Moderate
		Dyspepsia (10)	Antituberculosis	2	Probable (1), possible (1)	Definite (2)	Moderate
			Aspirin	7	Probable (4), possible (3)	Definite (7)	Mild (1), Moderate (6)
			Azithromycin	1	Probable	Definite	Moderate
		Hepatotoxicity (9)	Antituberculosis	2	Probable (1), possible (1)	Definite (1), not preventable (1)	Mild
			Atorvastatin	2	Probable (1), possible (1)	Definite (1), not preventable (1)	Mild
			Furosemide	3	Possible (2), doubtful (1)	Probable (1), not preventable (2)	Mild
			Omeprazole	1	Doubtful	Not preventable	Severe
			Paracetamol/Acetaminophen	1	Possible	Probable	Moderate
		Vomiting (6)	Antituberculosis	1	Probable	Definite	Moderate
			Aspirin	1	Probable	Definite	Moderate
			Amoxicillin/clavulanate	1	Possible	Definite	Moderate
			Enalapril	1	Possible	Definite	Moderate
			Hydrocortisone	1	Probable	Definite	Moderate
			Potassium chloride	1	Doubtful	Definite	Moderate
Hematologic	10 (8.1)	Bleeding (1)	Warfarin	1	Probable	Definite	Moderate
		Thrombocytopenia (9)	Aspirin	2	Possible (2)	Probable (2)	Mild
			Atorvastatin	2	Doubtful (2)	Probable (1), not preventable (1)	Mild
			Furosemide	3	Probable (1), possible (2)	Probable (2), not preventable (1)	Mild
			Unfractionated heparin	2	Possible (1), doubtful (1)	Probable (2)	Mild (1), severe (1)
Renal system	7 (5.7)	Acute kidney injury (7)	Furosemide	7	Probable (1), possible (5), doubtful (1)	Definite (3), probable (3), not preventable (1)	Moderate (6), severe (1)
Respiratory system	1	Tachypnea (1)	Furosemide	1	Possible	Probable	Moderate

ADRs—adverse drug reactions.

**Table 5:** Bivariate and multivariate logistic regression analysis predicting the occurrence of ADRs (N = 162).

Independent Variables	COR (95%CI)	*p* Value	aOR (95%CI)	*p* Value
Female sex	2.734 (1.126, 6.640)	0.026	2.119 (0.707, 6.354)	0.180
Alcohol consumption history	0.625 (0.315, 1.244)	0.181	0.675 (0.277, 1.646)	0.388
Traditional medicine use history	0.323 (0.118, 0.881)	**0.027**	0.286 (0.086, 0.957)	**0.042**
Number of diseases diagnosed	1.238 (1.011, 1.515)	**0.038**	0.855 (0.644, 1.136)	0.280
Disease of the endocrine system	2.436 (1.102, 5.390)	**0.028**	1.580 (0.550, 4.541)	0.395
Disease of respiratory system	1.455 (0.778, 2.719)	0.240	1.582 (0.632, 3.961)	0.327
Disease of the cardiovascular system	2.256 (1.139, 4.469)	**0.020**	1.10 (0.377, 3.204)	0.862
Disease of the digestive system	5.135 (1.088, 24.231)	**0.039**	8.784 (1.132, 68.127)	**0.038**
Symptoms, signs, or clinical findings, not elsewhere classified	3.378 (0.894, 12.768)	0.073	2.472 (0.511, 11.967)	0.261
Past medication	1.668 (0.894, 3.112)	0.108	1.227 (0.536, 2.810)	0.629
Number of inpatient medications	1.364 (1.170, 1.591)	**<0.001**	1.185 (0.954, 1.471)	0.125
A: Alimentary tract and metabolism	1.895 (1.011, 3.549)	**0.046**	1.776 (0.765, 4.123)	0.181
C: Cardiovascular system	3.75 (1.749, 8.039)	**0.001**	2.331 (0.619, 8.784)	0.211
J: Anti-infective for systemic use	1.979 (1.012, 3.869)	**0.046**	1.530 (0.576, 4.061)	0.394
N: Nervous system	1.780 (0.856, 3.70)	0.123	0.686 (0.233, 2.012)	0.492
R: Respiratory system	0.598 (0.265, 1.350)	0.216	0.595 (0.192, 1.846)	0.369
PIM use	6.783 (2.979, 15.445)	**<0.001**	4.747 (1.683, 13.389)	**0.003**

aOR—adjusted odds ratio; COR—crude odds ratio; PIM—potential inappropriate medication. Note: The bold highlighted figures under the ‘*p* value’ column indicate that the corresponding variables are significantly associated with incidence of ADR.

## Data Availability

The datasets used and/or analyzed during the current study are available from the corresponding author on reasonable request.

## Abbreviations

ADR	Adverse drug reactions
ADL	Activity of daily living
ALT	Alanine Aminotransferase
AOR	Adjusted odds ratio
ATC	Anatomical Therapeutic Chemical
AST	Aspartate Aminotransferase
ATC	Anatomical therapeutic chemical
BMI	Body mass index
CCI	Charlson comorbidity index
CNS	Central nervous system
COR	Crude odds ratio
GDS	Geriatric Depression Scale
ICD-11	International Classification of Diseases 11th Revision
IQ	Interquartile
IQR	Interquartile range
JMC	Jimma Medical Center
LMICs	Low-income countries
PIM	Potentially inappropriate medicine
TB	Tuberculosis
